# Acetylcholinesterase and Nicotinic Acetylcholine Receptors in Schistosomes and Other Parasitic Helminths

**DOI:** 10.3390/molecules22091550

**Published:** 2017-09-14

**Authors:** Hong You, Chang Liu, Xiaofeng Du, Donald P. McManus

**Affiliations:** 1 Molecular Parasitology Laboratory, Infectious Diseases Division, QIMR Berghofer Medical Research Institute, Brisbane, Queensland 4006, Australia; Chang.Liu@qimrberghofer.edu.au (C.L.); Xiaofeng.Du@qimrberghofer.edu.au (X.D.); 2 Parasitology Laboratory, School of Animal Medicine, Northeast Agricultural University, HarBin 150030, China

**Keywords:** schistosome, acetylcholinesterase, nicotinic acetylcholine receptors, drug targets, vaccine targets

## Abstract

Schistosomiasis, which is caused by helminth trematode blood flukes of the genus *Schistosoma*, is a serious health and economic problem in tropical areas, and the second most prevalent parasitic disease after malaria. Currently, there is no effective vaccine available and treatment is entirely dependent on a single drug, praziquantel (PZQ), raising a significant potential public health threat due to the emergence of PZQ drug resistance. It is thus urgent and necessary to explore novel therapeutic targets for the treatment of schistosomiasis. Previous studies demonstrated that acetylcholinesterase (AChE) and nicotinic acetylcholine receptors (nAChRs) play important roles in the schistosome nervous system and ion channels, both of which are targeted by a number of currently approved and marketed anthelminthic drugs. To improve understanding of the functions of the cholinergic system in schistosomes, this article reviews previous studies on AChE and nAChRs in schistosomes and other helminths and discusses their potential as suitable targets for vaccine development and drug design against schistosomiasis.

## 1. Introduction

Schistosomiasis, caused by parasitic flatworms of the genus *Schistosoma*, remains one of the most insidious and serious of the tropical parasitic diseases of clinical and public health significance. Currently, there is no effective vaccine to prevent schistosomiasis [1] and treatment relies heavily on a single drug, praziquantel (PZQ), despite the fact that it is ineffective in killing the intra-mammalian larval schistosomula stages and that PZQ drug resistance may emerge in the future in endemic areas because of its wide-spread use. Therefore, it is urgent and necessary to explore novel therapeutic targets for the treatment of schistosomiasis. Helminth parasites depend on fast-synaptic transmission in their neuromusculature to interact with the outside environment and respond to it. The neuromuscular system is targeted by a number of currently approved and marketed anthelminthics [2], including levamisole, pyrantel, monepantel [2,3] and metrifonate [4]. Inhibition of neuromuscular activity may lead to loss of muscle function and essential activities, including host attachment, feeding and mating, thereby interfering with parasite maturation [5], and, finally, parasite killing in the host. Metrifonate, targeting schistosome acetylcholinesterase (AChE) was shown to be effective in killing *Schistosoma haematobium* [6] but not *S. mansoni*; it was withdrawn from the World Health Organization’s (WHO) list of essential drugs for treating schistosomiasis [7] because of unacceptable toxicity to the host and the variable efficacy against different schistosome species [8]. 

**Figure 1:**
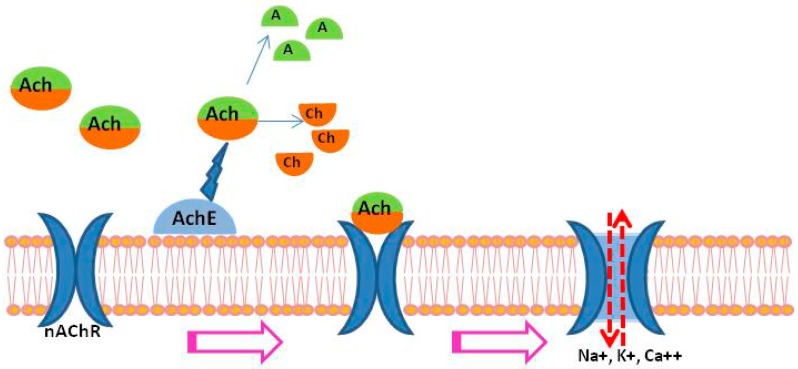
Predicted cholinergic signaling in schistosomes.

In the cholinergic system of flatworms, AChE plays an important role in regulating the interaction between acetylcholine (ACh) and the parasite nicotinic acetylcholine receptors (nAChRs) [9] by hydrolysing ACh to choline and acetate, allowing ions to pass down electro-chemical gradients into or out of cells [10] (Figure 1). The major role of AChE is termination of transmission at cholinergic synapses by rapid hydrolysis of the neurotransmitter, ACh, with high catalytic activity (at a turnover number of about 10,000/s) [11]. Signaling through cation-selective nAChRs, ACh mediates muscular contraction via membrane depolarization due to an influx of Na^+^, K^+^ or Ca^++^ which may produce clear effects on the worms, typically paralysis [12]. These processes are responsible for the long-term maintenance of low red-cell-membrane potential [13]. Based on a previous study showing exogenous cholinergic, agonists could cause flaccid paralysis of adult trematodes [14], a causal relationship was established between the activation of nAChR in *S. mansoni* muscle fibers and the flaccid paralysis caused by ACh in whole worms [15]. On the other hand, when AChE activity became inhibited, the higher levels of ACh caused excessive stimulation of nAChR leading to its desensitisation and closing of ion channels [9]. In mammalian cells, 17 known homologous subunits (α1–α10, β1–β4, γ, δ, and ε) assemble into different nAChR subtypes [16], including muscle nAChRs and neuronal nAChR subtypes. Muscle nAChRs are heteropentamers with a stoichiometry of α1_2_β1γδ subunits and are able to be activated by binding to ACh leading to a slow metabolic response. In contrast, neuronal nAChR subtypes are pentamers and are either homomers (α7, α8, α9 and α10) or heteromers of α and β subunits (α4β2, α3β4 and α4α2β3) and also of two different α subunits (α7α8, α9α10) [16]. Neuronal nAChRs are ligand-gated ion channels mediated by fast synaptic transmission. nAChRs contain two or more alpha subunits, and a dimer formed by the alpha subunits with adjacent subunit are critical for ACh binding. The transmembrane domain of each nAChR subunit consists of four helicoidal transmembrane domains (M1 to M4) [16]. The M2 domain of the nAChR subunits form the ion channel, which is only opened by the allosteric conformation change triggered by the binding of ACh. This channel is equally permeable to Na^+^ and K^+^, and Ca^++^ contributes approximately 2.5% to its total permeability [17]. New evidence has indicated direct coupling between G proteins and nAChRs in neurons, leading to a new hypothesis that binding to G proteins modulates the activity and signaling of nAChRs in cells, a process structurally associated with the opening of ligand-gated ion channels [18]. However, it remains to be determined how the binding between ACh and parasite AChRs activates AChRs, leading to opening of the ion channels. Given the studies on AChE and nAChRs in schistosomes and based on the research in mammalian cells mentioned above, the potential cholinergic signaling pathway predicted for schistosomes is shown in Figure 1.

The AChE of schistosomes, located on the surface of worms, breaks down host ACh in the blood into acetate (A) and choline (Ch), preventing overstimulation and blockage of the parasite AChRs. The AChRs of schistosomes having the same location as AChE may consist of pentameric proteins that form an ion channel embedded in the tegument of the parasites [19]. The ACh binds to the ligand binding domain (extracellular part) of nAChR subunits and the activated nAChR causes structural changes in the pentameric proteins, leading to the opening of the ligand-gated ion channels.

## 2. Acetylcholinesterase (AChE) in Schistosomes

AChE was first identified as a potential drug candidate in adult *S. mansoni* in 1952 by Bueding [20]. Further study showed that AChE is involved in the motor activity of *S. mansoni* [14], indicating that the cholinergic mechanisms are associated with neuromuscular function [21,22]. To date, AChE has been characterised from *S. mansoni*, *S. haematobium* and *S. bovis* [9,23], and *S. japonicum* [24], showing that the enzyme is present on the parasite tegument membrane and in the musculature, both in blood dwelling adults and schistosomula. Importantly, it was shown that AChE activity was highly enriched in the isolated external membrane [24,25] and its presence on the schistosome surface raised the possibility of a role other than termination of synaptic transmission and also suggested that it might prove to be an effective immunological target. A recent schistosome protein microarray study showed a predicted *S. japonicum* AChE precursor (AY810792) was significantly targeted by protective IgG1 immune responses in *S. haematobium*-exposed individuals that had acquired drug-induced resistance to schistosomiasis after PZQ treatment [26], thereby supporting consideration of AChE as a suitable anti-schistosomiasis vaccine candidate. Importantly, the absence of cross-reactivity with human AChE further supports schistosome AChE as a suitable target for immunological attack [22]. In vitro studies have shown that polyclonal anti-AChE antibodies are cytotoxic and cause complement-dependent killing 85% of schistosome parasites [22,27]. Most monoclonal antibodies raised in mice against *S. mansoni* AChE have been shown to interact only with parasite AChE, and not with the vertebrate enzyme, suggesting that the enzymes have different epitopes and that the specific schistosome AChE epitopes might be suitable candidates for drug and vaccine design [28].

Previous studies showed that circulating concentrations of ACh can result in an increase in glucose uptake in schistosomes in vitro [29]. Exposure of *S. haematobium* or *S. bovis*, but not *S. mansoni*, to low concentrations of ACh (10^−8^ to 10^−9^ M, the same concentration found in host blood) enhanced glucose uptake by the parasites, whereas at higher concentrations (10^−5^ to 10^−6^ M) ACh inhibited glucose uptake from host blood into the parasites. However, the influence of ACh on glucose uptake can also be reduced through inhibition of either tegumental AChE [30] or tegumental nAChR [9] of adult worms. It has been shown that the basal rate of glucose uptake in adult *S. haematobium* and *S. bovis* is about twice that in *S. mansoni* [29]. Indicative of the higher metabolic requirements for glucose in *S. haematobium* and *S. bovis*, relatively higher amounts of AChE activity are present on their teguments compared with *S. mansoni* [31]. These higher levels of AChE activity result in the recorded higher susceptibility to metrifonate [32], and this might explain why metrifonate mediates killing of *S. haematobium* but not *S. mansoni*. A recent report showed that in *S. japonicum* 90% of AChE activity occurs on the tegument [24], indicating the possibility of developing an effective inhibitor targeting AChE in *S. japonicum* as well. However, tegumental AChE could cause hydrolysis of local host glycogen stores or inhibit glycogen synthesis, either of which would induce more glucose available for the parasite in the local environment [33]. It also has been reported that AChE can increase by 60% glucose uptake by schistosomes from host blood by inhibiting the desensitisation of tegumental nAChR [29].

More recently, genomic studies of *S. mansoni* [34], *S. haematobium* [35] and *S. japonicum* [36] have stimulated further interest in the functional characterisation of cholinergic chloride channels and in revisiting the unusual inhibitory activity of AChE and the interaction between ACh and nAChRs in schistosomes. Previous studies and recent bioinformatics analyses identified different AChE amino acid sequences in *S. haematobium* and *S. japonicum*, but only a single AChE amino acid sequence in *S. mansoni* (Table 1)*.* However, further functional and structural studies of these different types of AChE in schistosomes need to be undertaken, as it is known that multiple isoforms of AChE occur in other parasites [37]. Indeed, early studies showed that two principal molecular forms (both globular) of AChE are present in *S. mansoni*, whereas very limited information is available on different isoforms of AChE in *S. japonicum* and *S. haematobium*. The two forms of *S. mansoni* AChE (*Sm*AChE), present in approximately equal amounts, with sedimentation coefficients of 6.5S and 8S [38], differ in their solubility characteristics and quaternary structure [39]. One form of *Sm*AChE is internalised in the muscle which interacts with heparin and is involved in cholinergic processes. The other form of *Sm*AChE is surface-localized and is anchored to the membrane via a covalently attached glycophosphatidylinositol (GPI) anchor and may be involved in non-cholinergic processes and in signal transduction [40]. This GPI-anchored AChE could be released from the schistosome surface membranes and attach to a PI-specific phospholipase C (PIPL-C), which can remove considerable quantities of AChE from the tegument of schistosomula in vitro, without impairing the viability of the parasite [41]. The released AChE was considered to trigger immediate replenishment of the surface enzyme. However, this process does occur with another GPI-anchored protein, alkaline phosphatase, which is also present on the schistosome surface [27].

## 3. Nicotinic Acetylcholine Receptors (nAChRs) in Schistosomes

As indicated earlier, the neuromuscular effects of acetylcholine are typically mediated by gated cation channels of the nicotinic receptor (nAChR) family within the nervous system of parasites. Bentley et al. (2003) identified and reported (https://www.ncbi.nlm.nih.gov/pubmed/) the sequences of two types of nAChRs from *S. mansoni*, including nAChR alpha subunit precursor (AAR84361) and nAChR non-alpha subunit precursor (AAR84362) but undertook no further characterization of these molecules. Recent bioinformatics analyses identified numerous nAChR subunits in the *S. mansoni* genome and showed that approximately half of these subunits represented a motif involved in chloride-selectivity [42]. These putative *S. mansoni* acetylcholine gated chloride channels (*Sm*ACCs) form a unique clade within the larger family of nAChRs [42]. Two types of *Sm*ACC (*Sm*ACC-1 and 2) have been characterized from *S. mansoni*, both of which localize to regions of the peripheral nervous system that innervate the body wall muscles. RNA interference, targeting 5 putative nAChR subunits (Smp_155790, Smp_0.37960, Smp_132070, Smp_176310 (*Sm*ACC-1) and Smp_142690 (*Sm*ACC-2); Table 1), was used to knockdown nAChRs in larval schistosomula, and this resulted in 90% and 60% reductions in the transcription levels of *Sm*ACC-1 and 2, respectively. Furthermore, *Sm*ACC-1 has been shown to play an important role in forming a functional homomeric chloride channel and is activated selectively by a panel of cholinergic agonists [42]. This novel clade of nicotinic chloride channels may act as inhibitory modulators of schistosome neuromuscular function.

Three types of nAChRs have been isolated and partially characterized from *S. haematobium*, namely *Sh*AR1α (AAR84357) [19,43], *Sh*AR1β (AAR8435) [19] and *Sh*AR2β (AAX59989) [44]. *Sh*AR1α is located on the parasite surface and may contribute to the potentiation of the uptake of glucose from host blood in response to circulating concentrations of ACh [19]. AChE and nAChR are both found to be predominantly concentrated on the dorsal surface of adult male *S. haematobium* [43], with only low representation on the male head, tail, and ventral surface and gynaecophoric canal of the males and the entire female tegument [45]. 

Recently, additional putative nAChR subunits have been identified from *S. mansoni* [34], *S. haematobium* [35] and *S. japonicum* [36] (Table 1), but no further functional characterization of these components has been undertaken. The motif analysis (http://www.genome.jp/tools/motif/), that we have undertaken, indicates a highly conserved neurotransmitter-gated ion-channel ligand binding domain and transmembrane region present in the majority of these schistosome nAChR subunits. Both extracellular and intracellular domains occur in the nAChRs of *S. haematobium* (AAR84357 *, AAR84358 *, AAX59989 *, B_00805, A_02378), *S. japonicum* (Sjp_0034800 #, Sjp_0131150 and Sjp_0015560) and *S. mansoni* (AAR84361 *, AAR84362 *, Smp_139330, Smp_031680, Smp_142700, Smp_180570, Smp_132070.1, Smp_132070.2, Smp_012000, Smp_037960, Smp_157790, Smp_142690, Smp_176310). A conserved Cys-loop motif is present in the neurotransmitter-gated ion-channel ligand binding domain of the majority of the *S. haematobium, S. mansoni* and *S. japonicum* nAChR subunits as shown in Table 2 and suggests the schistosome nAChRs are members of a Cys-loop ligand-gated ion channel superfamily where the Cys-loop is a 13 amino acid sequence linked by a cysteine disulfide bond contained within the extracellular domain. This Cys-loop domain plays an important role in the structural binding between ACh and nAChR [46] and may be involved in inhibitory amine neurotransmission in parasites [47].

## 4. AChE and AChRs in Other Helminths

AChE has been identified in a number of other helminth parasites [48]. ACh and AChE inhibitors relax worm musculature, decrease worm motility and eventually produce paralysis in, for example, *Fasciola hepatica* [49], *Dipylidium caninum* [48] and the nematode, *Nippostrongylus brasiliensis* [50]. An early study showed that *N. brasiliensis* actively secretes AChE during infection and that this enzyme is able to induce an immune response in infected rats [51]. More recent research indicated that one of the potential functions of AChE in *Trypanosoma musculiv* is to alter the host cytokine environment and depress the development of M2 macrophages which are deleterious to worm survival [52]. AChE activity has also been detected in the cell bodies and extracellularly in the neuropile of the cerebral ganglia of adult *F. hepatica* [53]. Further, that study showed an AChE reaction product associated with synaptic endings that localized at the site of synaptic contact between the zone of apposition of the pre- and postsynaptic terminals, suggesting an important role in synaptic transmission in this liver fluke [53]. Changes in the molecular forms and activity of AChE during different life cycle stages in parasites may reflect the presence of a complex nervous system, a situation apparently evident in the cyclophyllidean cestode, *Mesocestoides corti*, during development of the reproductive apparatus in segmented and adult worms, which opens up the possibility of developing an anti-AChE intervention as an effective therapeutic strategy against this and other tapeworms [54].

AChRs have received considerable recent attention as potential anthelmintic drug targets in nematodes. AChRs represent major targets for cholinergic agonist or antagonist anthelmintic drugs such as levamisole, pyrantel, tribendimidine and derquantel [55]. Despite the wide diversity of nAChRs present in nematodes, which are located not only at the neuromuscular junction but also in the nerve ring and in the pharynx [56], only a few receptor subtypes have so far been characterized. The model free living nematode, *Caenorhabditis elegans*, has been shown to possess two stimulatory AChRs, one sensitive to levamisole (L-AChR) and the other sensitive to nicotine (N-AChR) [57]. The latter is a homopentamer of the ACR-16 subunit in *C*. *elegans* [58], which is highly conserved in a variety of other parasitic nematode species representing different clades. In contrast, L-AChR is composed of five subunits including three α-subunits (unc38, unc-63, and lev-8), and two non-α-subunits (unc-29 and lev-1) [59]. The parasitic nematodes have lost specific subunits and functional receptors, and with different pharmacology, have been produced with different subunit combinations in each species [60]. An analysis of genomic data from 25 nematode parasite species indicated multiple independent duplications of unc-29 as positive, directional selections acting on amino acid positions associated with subunit assembly. It has been demonstrated that functional divergence in AChRs with novel pharmacology occurs more rapidly and may be mediated by alteration of receptor assembly, providing a foundation for understanding the broader context of changing anthelmintic drug targets across the parasitic nematodes [61]. Furthermore, identification of four gene copies of AChRs from the sheep nematode, *Haemonchus contortus*, also demonstrated that each copy has acquired unique functional characteristics [61].

Functional AChRs have been reported in the pig nematodes *Ascaris suum* and *Oesophagostomum dentatum*, and in *H. contortus* Muscle movement in *A. suum* is driven by activation of the nAChRs that are found synaptically and extra-synaptically over the surface of the muscle cells. In vitro expression in Xenopus oocytes of two of the *A. suum* AChR subunits [62], which share similar sequence to unc-29 and unc-38 in *C. elegan*, showed that changing the expression level of a single receptor subunit dramatically modified the effectiveness of a number of anthelmintics drugs. This information may prove useful in the future development of screens to identifying novel anti-parasite compounds.

Three subtypes of AChR isolated from *A. suum* have been named as N (for nicotine), L (for levamisole) and B (for bephenium) [12]. In vitro reconstitution experiments showed that receptors with properties similar to those of the ‘N’ and ‘L’ subtypes could be produced by expression of the Asu-unc-29 and Asu-unc-38 subunits [62]. Changing the ratio of the two subunits resulted in differences in sensitivity to both the anthelmintics pyrantel and oxantel, demonstrating that the latter acts on the ‘N’ AChR and the former is more active on the ‘L’ sub-type [63]. However, the lev-8 and lev-1 genes are not detectable in *H. contortus*. A study on in vitro expression of *H. contortus* levamisole-sensitive receptors (acr-8, unc-29, unc-38 and unc-63 subunits) in *Xenopus* oocytes [64] showed that the increased expression of receptors lacking acr-8 might be associated with levamisole resistance [65]. The two well-conserved AChR subunits, unc-38 and unc-29, identified in H. contortus and A. *Suum* play important roles in rescuing levamisole-resisteance [66].

Four biophysical subtypes of AChR (Ode-unc-38, Ode-unc-63, Ode-unc-29 and Ode-acr-8) have been identified in *Oesophagostomum dentatum* nAChR and expressed functionally in *Xenopus* oocytes [60]. The results obtained further supported previous studies showing that a differential combination of these AChRs subunits can affect sensitivity and resistance to the cholinergic anthelmintics pyrantel, tribendimidine and derquantel.

## 5. Conclusions

AChE and nAChRs play important roles in the neuromuscular and other signaling systems of parasitic helminths and are targets of a number of currently marketed anthelminthics. AChE is an important metabolic enzyme in schistosomes present in the musculature and on the surface of the intra-mammalian blood stages where it has been implicated in the modulation of glucose scavenging from host blood. Targeting schistosome AChE, metrifonate was used historically as an effective drug for the treatment of urinary schistosomiasis, strongly emphasising the important role of the enzyme in the neuromuscular or cholinergic systems. Subsequent studies in the 1980/90s on AChE in *S. haematobium* and *S. mansoni* revealed further the neuromuscular effects of AChE through studies inhibiting the interaction between ACh and the nAChR subunits regulating the gated cation channels within the nervous system of both parasites. Three types of nAChRs were initially partially characterized from *S. haematobium* with two nAChRs isolated from *S. mansoni* [19,43,44]. Characterisation of ACh-gated chloride channels (*Sm*ACCs) in *S. mansoni* followed in 2014 [42]. These studies emphasised the important role of nAChRs in inhibitory neurotransmission in schistosomes and their potential as major targets for cholinergic agonists. However, our limited understanding of the neuromuscular and cholinergic systems in the blood flukes has hampered the development of effective drugs targeting these pathways. Given the availability of the extensive genomic data for the three major clinically relevant schistosome species now available and recent research advances on AChE/AChRs in other helminths, future research can be expected to result in the discovery of novel drug or vaccine candidates targeting these critical components.

## Figures and Tables

**Table 1:** AChEs and nAChRs identified in *S. haematobium*, *S. mansoni* and *S. japonicum*.

Schistosome Species	AChE	nAChR				
	Protein ID	Description	Protein ID	Conserved Domains/Motifs		
				Ligand Domain	Trans-Membrane Region	Cys-Loop
*S. haematobium*	A_03825	AChR 1alpha	AAR84357 *	√	√	√
	KGB33661 * (XP_012793429 *)	AChR 2beta	AAR84358 *	√	√	√
		AChR 2beta	AAX59989 *	√	√	√
	KGB33101 * (XP_012792873 *; A_04487 ^~^)	nAChR beta 3 (Dbeta 3) subunit	A_01504 ^~^			
			A_01761 ^~^			√
	KGB37011 * (XP_012796773 *; A_02007 ^~^)	Putative nAChR alpha 9b subunit	B_00805 ^~^	√	√	
		AChR beta, putative	A_05298 ^~^	√		
	AAO49838 * (AAO62355 *, AAQ14322 *)	AChR-related	A_06497 ^~^	√		√
		ACh-gated chloride channel-1 (ShACC-1)	A_02378 ^~^	√	√	√
		ACh-gated chloride channel-2 (ShACC-2)	A_06346 ^~^			
***S. mansoni***	AAQ14321 * (CCD58664 * XP_018645024 *)	AChR alpha subunit precursor	AAR84361 *			√
		AChR non-alpha subunit precursor	AAR84362 *			√
		ACh-gated chloride channel-1 (SmACC-1)	Smp_176310 ^#^	√	√	√
		ACh-gated chloride channel-2 (SmACC-2)	Smp_142690 ^#^	√	√	√
		Putative nAChR alpha 9b subunit	Smp_135040 ^#^			
		AChR-related	Smp_012000 ^#^	√	√	√
		Putative nAChR subunit	Smp_101990 ^#^			
			Smp_037960 ^#^	√	√	√
			Smp_157790 ^#^	√	√	√
			Smp_132070 ^#^			√
			Smp_180570 ^#^	√	√	√
			Smp_197600 ^#^	√		√
			Smp_142700 ^#^	√	√	
			Smp_031680 ^#^	√	√	√
			Smp_139330 ^#^	√	√	√
***S. japonicum***	Sjp_0045440 ^#^	Putative nAChR alpha 9b subunit	Sjp_0071780 ^#^			
			Sjp_0034800 ^#^	√	√	√
		Neuronal AChR subunit alpha-7	Sjp_0082390 ^#^	√		√
	Sjp_0070510 ^#^	AChR-related	Sjp_0131150 ^#^	√	√	√
	Sjp_0036280 ^#^	Putative nAChR subunit	Sjp_0015560 ^#^	√	√	
			Sjp_0066940 ^#^		√	
	ANH56887 [24] *	ACh-gated chloride channel (SjACC-1)	Sjp_0115170 ^#^			

Note: * protein ID cited from PubMed (https://www.ncbi.nlm.nih.gov/pubmed); ^#^ cited from GeneBD (http://www.genedb.org/Homepage); ~ cited from WormBase Parasite (http://parasite.wormbase.org/index.html). Amino acid sequences of all proteins listed in this Table can be found in Supplementary Materials Table S1.

**Table 2:** Cys-loop motif sequences present in the nAChR subunits of *S. haematobium*, *S. mansoni* and *S. japonicum.*

Schistosome Species	nAChR	Cys-Loop Sequence
*S. haematobium*	AAR84357	CNIDILWFPFDEQSC
	AAR84358	CDIEVNWFPFDSQNC
	AAX59989	CQVEITYFPFDSQVC
	A_01761	CSVDIKYFPFDRQKC
	A_06497	CPLDVSFFPFDYQTC
	A_02378	CPVKIKYFPYDKQVC
*S. mansoni*	AAR84362	CDIEVNWFPFDSQNC
	Smp_139330	CDIEVNWFPFDSQNC
	AAR84361	CNIDILWFPFDEQSC
	Smp_031680	CNIDILWFPFDEQSC
	Smp_197600	CKIDITYFPFDDQSC
	Smp_157790	CKIDIKSFPFDEQTC
	Smp_132070.1	CPIDIKNFPFDYQHC
	Smp_132070.2	CPIDIKNFPFDYQHC
	Smp_012000	CPLDVSFFPFDYQTC
	Smp_142690	CQVEITYFPFDSQVC
	Smp_037960	CEVEITYFPFDTQIC
	Smp_176310	CPVKIKYFPYDKQVC
	Smp_180570	CQVDITLFPFDQQNC
*S. japonicum*	Sjp_0034800	CNVDVLYFPFDHQLC
	Sjp_0131150	CPLDVSFFPFDYQTC
	Sjp_0082390	CDIEVNWFPFDSQNC

Note: The Cys-loop sequence is shown with the cysteine residues and conserved FP-D-Q highlighted in black shading while gray shading indicates conserved residues.

## Supplementary Materials

Supplementary materials are available online. Table S1: Amino acid sequences of AChEs and nAChRs identified in *S. haematobium*, *S. mansoni* and *S. japonicum*.

## Abbreviations

The following abbreviations are used in this manuscript

PZQ	praziquantel
AChE	acetylcholinesterase
ACh	acetylcholine
nAChRs	nicotinic acetylcholine receptors
GPI	glycophosphatidylinositol
PIPL-C	PI-specific phospholipase C
*Sm*ACCs	*S. manosni* acetylcholine gated chloride channels
*Sh*AR1α	*S. haematobium* nicotinic acetylcholine receptor 1 α
*Sh*AR1β	*S. haematobium* nicotinic acetylcholine receptor 1 β
*Sh*AR2β	*S. haematobium* nicotinic acetylcholine receptor 2 β
